# Diffusion MRI data, sulcal anatomy, and tractography for eight species from the *Primate Brain Bank*

**DOI:** 10.1007/s00429-021-02268-x

**Published:** 2021-07-15

**Authors:** Katherine L. Bryant, Dirk Jan Ardesch, Lea Roumazeilles, Lianne H. Scholtens, Alexandre A. Khrapitchev, Benjamin C. Tendler, Wenchuan Wu, Karla L. Miller, Jerome Sallet, Martijn P. van den Heuvel, Rogier B. Mars

**Affiliations:** 1 Wellcome Centre for Integrative Neuroimaging, Centre for fMRI of the Brain (FMRIB), Nuffield Department of Clinical Neurosciences, John Radcliffe Hospital, University of Oxford, Headington, Oxford, OX9 3DU UK; 2 Department of Complex Trait Genetics, Centre for Neurogenomics and Cognitive Research, Amsterdam Neuroscience, Vrije Universiteit Amsterdam, Amsterdam, The Netherlands; 3 Wellcome Centre for Integrative Neuroimaging, Department of Experimental Psychology, University of Oxford, Oxford, UK; 4 Department of Oncology, University of Oxford, Cancer Research UK and Medical Research Council Oxford Institute for Radiation Oncology, Oxford, UK; 5 Univ Lyon, Université Lyon 1, Inserm, Stem Cell and Brain Research Institute U1208, 69500 Bron, France; 6 Department of Clinical Genetics, Amsterdam Neuroscience, Amsterdam UMC, Vrije Universiteit Amsterdam, Amsterdam, The Netherlands; 7 Donders Institute for Brain, Cognition and Behaviour, Radboud University Nijmegen, Nijmegen, The Netherlands

**Keywords:** Comparative, Connectivity, Cortex, Anthropoid elaboration, Optic radiation, Cingulum bundle

## Abstract

Large-scale comparative neuroscience requires data from many species and, ideally, at multiple levels of description. Here, we contribute to this endeavor by presenting diffusion and structural MRI data from eight primate species that have not or rarely been described in the literature. The selected samples from the Primate Brain Bank cover a prosimian, New and Old World monkeys, and a great ape. We present preliminary labelling of the cortical sulci and tractography of the optic radiation, dorsal part of the cingulum bundle, and dorsal parietal–frontal and ventral temporal-frontal longitudinal white matter tracts. Both dorsal and ventral association fiber systems could be observed in all samples, with the dorsal tracts occupying much less relative volume in the prosimian than in other species. We discuss the results in the context of known primate specializations and present hypotheses for further research. All data and results presented here are available online as a resource for the scientific community.

## Introduction

The comparative method is one of the primary ways to gain insight into brain evolution. In the study of humans and other primates, this paradigm has been very successful in elucidating the evolution of the size of the brain and some its constituent parts [e.g., Miller et al. (2019); Smaers and Vanier (2019)]. Although these methods are able to identify specializations in different branches of the primate tree, including instances of mosaic evolution (Barton and Harvey 2000; Smaers and Soligo 2013), a poverty of data in this field has also meant that few datasets are available and definition of constituent parts is often difficult to determine, giving rise to strong controversies in the field. A prominent example of such a debate is whether the size of human prefrontal cortex follows trends expected based on other primates (Barton and Venditti 2013; Passingham and Smaers 2014). Moreover, the focus on size or relative size of the brain or its parts obscures the fact that many other changes can have occurred across different lineages, including changes in the connections of the brain and in the number of areas (Krubitzer and Kaas 2005; Mars et al. 2017). However, the labor intensive and destructive nature of many of the traditional techniques to study such changes makes them unfeasible for large-scale comparisons.

The rise of neuroimaging as a tool in comparative neuroscience over the last decade has the potential to address these problems (Mars et al. 2014). Neuroimaging techniques such as magnetic resonance imaging (MRI) have the advantage that the brains can be scanned in whole, potentially using different sequences, without destroying or damaging the tissue (Thiebaut De Schotten et al. 2019). This means that tissue that has been scanned remains available for further analyses, whether using neuroimaging or more traditional techniques. The digital nature of neuroimaging data makes data sharing easier, facilitating direct comparisons across various datasets. As neuroimaging and traditional techniques are often the expertise of different labs, this type of data sharing is crucial to reach a full understanding of brain diversity. Neuroimaging thus has potential beyond producing new data; it allows a fusion of data types describing brain organization at different levels of resolution (McKavanagh et al. 2019). This, in turn, enhances understanding of brain diversity at multiple scales.

Diffusion MRI in particular has recently proven a popular tool in comparative neuroimaging. Tractography algorithms allow one to use these data to reconstruct the major fiber bundles of the brain, which are an excellent starting point for quantitative comparisons of brain organization. Pioneering work on comparative tractography identified the arcuate fascicle, one of the major tracts connecting areas involved in language processing in the human brain, as expanded in the human as compared to the macaque and chimpanzee brain (Rilling et al. 2008). Subsequent work has investigated this result in the light of other changes between these brains (Ardesch et al. 2019; Eichert et al. 2019) and embarked on systematic comparisons of tracts between human and non-human brains (Hecht et al. 2015; Mars et al. 2016; Takemura et al. 2017; Bryant et al. 2019). The creation of whole-brain tract libraries for various species (Thiebaut de Schotten et al. 2012; Bryant et al. 2020; Warrington et al. 2020), advances in data processing (Li et al. 2013; Mars et al. 2019), and integration with other modalities (Eichert et al. 2020) increasingly open up new avenues for novel comparisons of brain organization.

To facilitate this endeavor, we here present high-quality structural and diffusion MRI data from eight primate species, distributed across the different branches of the primate tree. All samples were acquired from the Primate Brain Bank (PBB), a non-profit initiative at the Netherlands Institute for Neuroscience that collects brain samples from deceased primates from a number of zoos in the Netherlands (Kaas and van Eden 2011). We also present labelling of sulcal anatomy based on reconstructions of the brains’ cortical surfaces and tractography of some of the major white matter fiber bundles using standardized protocols. All data and results are available online to the scientific community.

## Materials and methods

### Samples and scanning

**Fig. 1:**
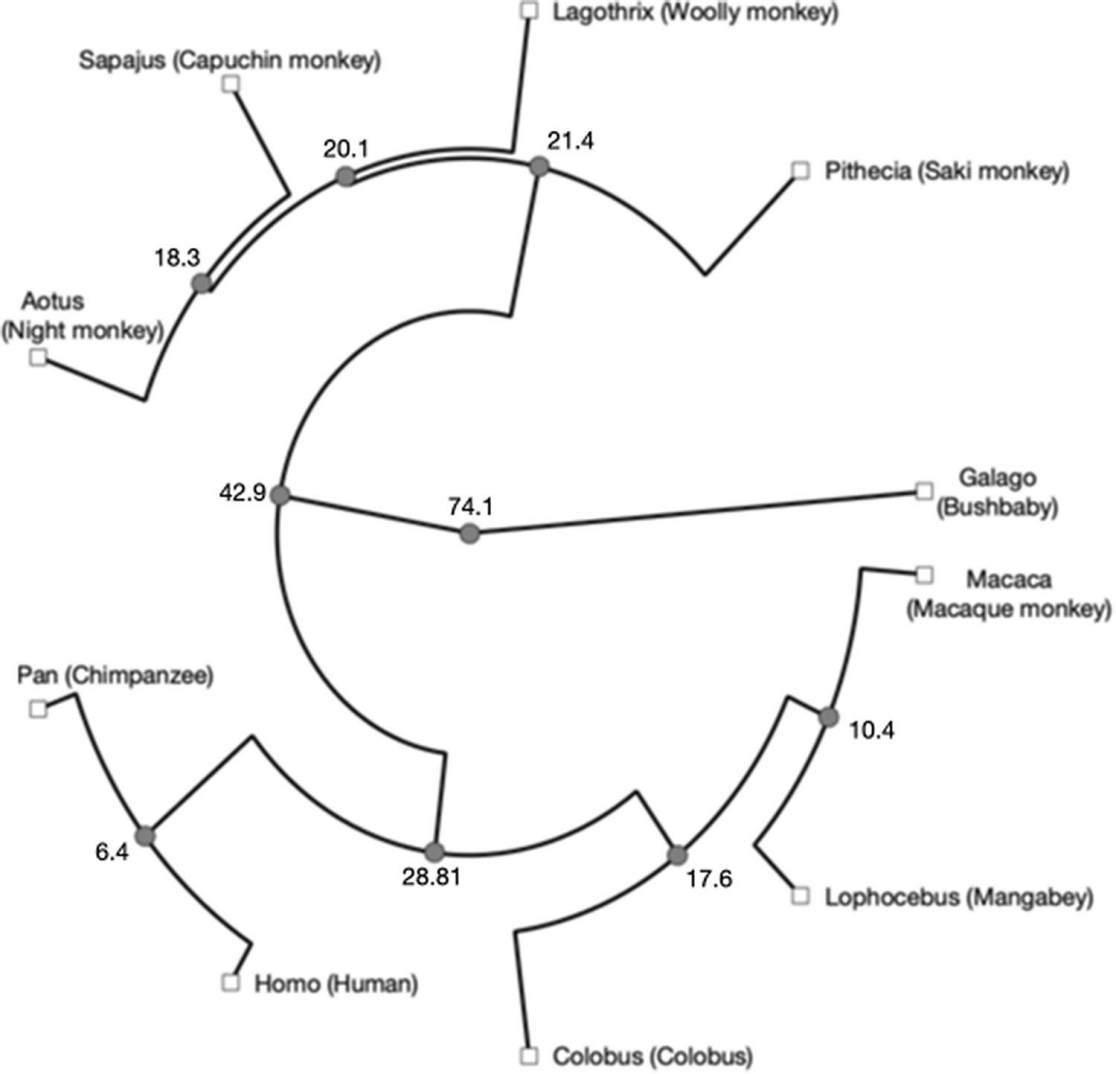
Phylogenetic relationships between the species in this study. Numbers indicate median divergence times (in millions of years) based on genomic data (Hedges et al. 2015)

We acquired eight brains from the Primate Brain Bank (PBB)—one prosimian primate (bushbaby), four New World monkeys (night monkey, woolly monkey, white-faced saki, and tufted capuchin), two Old World monkeys (black-and-white colobus and grey-cheeked mangabey), and one great ape (chimpanzee). Demographics are listed in Table 1. All samples were returned to the PBB after scanning and remain available for future research. In addition, we used data from one rhesus macaque (*Macaca mulatta*, male, 4.03 years of age at death) from a previous study (Folloni et al. 2019) for comparison with this most commonly studied primate. Phylogenetic relationships between all species in this study and the human are displayed in Fig. 1.

**Table 1:** Demographic information of the scanned PBB samples

Latin name	Common name	Sex	Age (years)	PBB identifier
Prosimians				
* Galago senegalensis*	Senegal bushbaby	M	20.9	PB0123
New world monkeys				
* Pithecia pithecia*	White-faced saki	M	4	PB0720
* Lagothrix lagotricha*	Woolly monkey	F	8	PB0630
* Sapajus apella*	Tufted capuchin	M	22	PB0737
* Aotus lemurinus*	Night monkey	M	15.3	PB0425
Old world monkeys				
* Colobus guereza*	Black-and-white colobus	M	23.7	PB0113
* Lophocebus albigena*	Grey-cheeked mangabey	F	27	PB0714
Great apes				
* Pan troglodytes*	Chimpanzee	F	54	PB1301

Prosimian and monkey data were acquired locally on a 7 T magnet with an Agilent DirectDrive console (Agilent Technologies, Santa Clara, CA, USA) using a 2D diffusion-weighted spin-echo protocol with single line readout (DW-SEMS, TE/TR: 25 ms/10 s; Matrix = 128 × 128; number of slices: 128; Resolution: 0.3 × 0.3 × 0.3 mm for the galago, 0.4 × 0.4 × 0.4 mm for the night monkey, 0.5 × 0.5 × 0.5 mm for the saki monkey, 0.6 × 0.6 × 0.6 mm for the woolly, capuchin, colobus, and mangabey; diffusion data were acquired over the course of ~ 52.5 h). 16 non-diffusion-weighted (*b* = 0 s/mm^2^) and 128 diffusion-weighted (*b* = 4000 s/mm^2^) volumes were acquired with diffusion directions distributed over the whole sphere. The brains were soaked in PBS before scanning and placed in fluorinert during the scan.

Great ape data were acquired on a 7 T whole-body scanner with an 28 channel knee coil (QED) using a 3D diffusion-weighted steady-state free precession (McNab et al. 2009) protocol (DW-SSFP, TE/TR: 18 ms/22 ms; flip angle: 50°; resolution: 0.6 × 0.6 mm^2^; number of slices: 176; slice thickness: 0.6 mm; EPI factor: 1; diffusion data were acquired over the course of ~ 50 h). 12 non-diffusion-weighted (*q*-value = 20 cm^−1^) and 240 diffusion-weighted (*q*-value = 300 cm^−1^) volumes were acquired with diffusion directions distributed over the whole sphere. Accurate modelling of DW-SSFP data requires the estimation of DW-SSFP dependencies (T1, T2 and B1) (Buxton 1993). To account for this, T_1_-weighted data were obtained using a turbo inversion recovery (TIR, TIs: 30, 60, 120, 240, 480 and 940 ms; TE/TR: 17 ms/1020 ms; resolution: 0.8 × 0.8 mm^2^; number of slices: 112; slice thickness: 0.8 mm; TIR data acquired over the course of ~ 2 h), T_2_-weighted data were obtained using a turbo spin-echo (TSE, TEs: 8.7, 17, 26, 35, 52 and 70 ms; TR: 1000 ms; resolution: 0.8 × 0.8 mm^2^; number of slices: 112; slice thickness: 0.8 mm; TSE data acquired over the course of ~ 2 h) and B_1_ maps were estimated using an actual flip angle imaging (Yarnykh 2007) sequence (AFI; TE: 2.6 ms; TRs: 7.7 and 21 ms; resolution: 3 × 3 mm^2^; number of slices: 32; slice thickness: 3 mm; AFI data acquired in ~ 43 s).

MGE data for surface reconstruction for the prosimian and monkey samples from the PBB were also acquired on a 7 T magnet with an Agilent DirectDrive console (Agilent Technologies, Santa Clara, CA, USA) using a 3D multi-gradient-echo sequence (MGE3D, TE1/δTE/TR = 7.5 ms/11 ms/98 ms; resolution = 0.15 × 0.15 × 0.15 mm (galago), 0.2 × 0.2 × 0.2 mm (night monkey), 0.25 × 0.25 × 0.25 mm (saki monkey), 0.3 × 0.3 × 0.3 mm (other samples); data were acquired over 1 h).

Structural scans for the great ape were acquired on a 7 T whole-body scanner with a 28 channel knee coil (QED) using a 3D true fast imaging with steady state free precession protocol (TRUFI, TE/TR: 5.06 ms/10.12 ms; flip angle: 39°; resolution = 0.4 × 0.4 mm^2^; number of slices: 256; slice thickness: 0.4 mm; TRUFI data acquired over the course of ~ 22 min). 4 TRUFI datasets were acquired (phase increment: 0°, 90°, 180°, and 270°) and averaged via root sum of squares.

### Diffusion MRI data preprocessing

All prosimian and monkey datasets were converted to NIFTI format, reoriented to approximate AC/PC orientation with the origin set at the middle of the anterior commissure, and preprocessed using tools from FSL (www.fmrib.ox.ac.uk/fsl). These steps are implemented in the ‘phoenix’ module of the MR Comparative Anatomy Toolbox (Mr Cat; www.neuroecologylab.org). We used BedpostX to fit a crossing fiber model to each dataset (Behrens et al. 2007), allowing up to three fiber orientations per voxel. This tool produces voxel-wise posterior distributions of fiber orientations that are subsequently used for probabilistic tractography.

For the great ape DW-SSFP data, prior to analysis a Gibbs-ringing correction (Kellner et al. 2016) was performed on both the diffusion-weighted and non-diffusion-weighted datasets. To account for the DW-SSFP dependencies (T_1_, T_2_ and B_1_), T_1_ and T_2_ maps were estimated from the TIR and TSE datasets assuming mono-exponential signal evolution (the Gibbs correction was also performed on the TIR and TSE data). A B_1_ map was generated from the AFI data following the methodology described in Yarnykh (2007). All co-registrations between and within modalities were performed using FSL FLIRT using a 6 degrees-of-freedom (rotation and translation) transformation. These datasets were subsequently fit using custom diffusion tensor and ball and two stick models which incorporates the full Buxton DW-SSFP signal equations (Buxton 1993) using cuDIMOT (Hernandez-Fernandez et al. 2019).

### Surface reconstruction

Cortical surfaces were reconstructed from the structural scans using FreeSurfer v6.0 (Fischl 2012). Processing steps included tissue segmentation of grey and white matter, identification of subcortical structures, and surface reconstruction. To obtain successful reconstructions of the non-human primate brains, the FreeSurfer pipeline was complemented with tools from FSL v6.0.1 (Jenkinson et al. 2012), ANTs (Avants et al. 2011), and MATLAB (2017a, The Mathworks, Inc., Natick, Massachussets, United States).

A brain mask was used to separate brain tissue from any other structures present in the scans such as fiducial markers. Bias field correction was then applied to the images with ANTs. Voxel intensities from the original MGE and TRUFI images were inverted to obtain a T1-like contrast with low voxel intensities in gray matter voxels and high intensities in white matter voxels. For the mangabey, an average b0 volume was used as the original image instead. Next, tissue segmentation was performed using FreeSurfer. Manual corrections were made where necessary to correct segmentations of white matter, cerebellum, and subcortical structures. FreeSurfer’s default Talairach registration step was complemented by a step-by-step registration process, starting with a registration from the subject at hand to a macaque template brain (Seidlitz et al. 2018), followed by a registration from the macaque template to a chimpanzee template brain (based on data from chimpanzeebrain.org), and from the chimpanzee template to human Talairach space. The chimpanzee brain was first registered to the chimpanzee template brain and then to human Talairach space, skipping the macaque template step. This step-by-step registration process ensured that major brain structures such as the cerebellum and thalamus were correctly segmented in the smaller brains. Next, surface reconstruction was started on the high-resolution volumes. The pial surface was refined by alignment to a downsampled version of the original volumes using the high-resolution scripts from the HCP FreeSurfer pipeline (https://github.com/Washington-University/HCPpipelines/tree/master/FreeSurfer/custom). Tissue segmentations and surface reconstructions were visually checked for accuracy and consistency across datasets. It should be noted that the reliance on manual interference does produce some artifacts, especially in the territory underneath the corpus callosum. This subcortical area, however, is usually masked out when working with cortical surface data.

Surfaces were converted to GIFTI format and a midthickness surface was created by averaging the pial and white matter border surfaces. For display purposes, the midthickness surface was smoothed (strength 0.5, 15 iterations) using the Workbench Command tools of Connectome Workbench (Marcus et al. 2011).

### Tractography

Tractography recipes were defined using the conventions of XTRACT, a recently released tool to standardize tractography across species (Warrington et al. 2020). Each tract was defined through a seed and a target or waypoint mask, a set of exclusion masks, and potentially a stop mask. All recipes were drawn with respect to homologous sulci and gyri or as similar a location as possible. All recipes are available online (see "Data availability" statement). These tracts give a good balance between association and non-association fibers and fibers crossing through areas with and without crossing fibers.

#### Optic radiation (OR)

The optic radiation consists of fibers from the lateral geniculate nucleus (LGN) of the thalamus to the primary visual cortex. It was seeded in the LGN and the target mask consisted of a coronal plane just anterior to the lunate sulcus. Exclusion masks consisted of an axial block of the brainstem, a coronal block of fibers directly posterior to the LGN to select fibers that curl around dorsally, and a coronal plane anterior to the seed to prevent leaking into longitudinal fibers.

#### Cingulum bundle, dorsal segment (CBD)

The dorsal segment of the cingulum bundle was seeded just at the front of the posterior third of the corpus callosum and had a target at the start of the genu of the corpus callosum. A mid-sagittal exclusion mask prevented contralateral tracts and a coronal mask through the territory of the superior longitudinal fasciculi at the level of the midpoint of the corpus callosum prevented leakage into this system. Finally, an axial exclusion mask below the corpus callosum ensured that we only picked up tracts belonging to the dorsal segment of the cingulum bundle. Tractography was run with the seed and target masks and again with the roles of the two masks reversed and the results were averaged.

#### Superior longitudinal fascicle complex (SLFc)

The three branches of the superior longitudinal fasciculus are reconstructed together using an extension of the approach taken by Thiebaut de Schotten et al. (2011). In each case, a coronal plane in the region of the central sulcus (if present) is used as a seed along with two target masks. Frontally, a target mask through the territory of the SLFc was placed at the level of the anterior commissure. Posteriorly, a large coronal target mask in the parietal cortex, was placed immediately posterior to the margin of the cingulate gyrus, if present. Exclusion masks were placed in the mid-sagittal plane, axially to exclude tracts running ventrally of parietal–frontal cortex, and through the cingulum bundle at the level of each seed and target mask.

#### Longitudinal fronto-temporal tract (LFT)

A ventral longitudinal tract, often termed inferior fronto-occipital fascicle (IFOF) or extreme capsule fiber complex, has been identified using tractography in the human (Forkel et al. 2014), and more recently in great apes and monkeys (Mars et al. 2016; Roumazeilles et al. 2020). It courses from ventral frontal cortex, through the extreme capsule, running medially through the temporal cortex, reaching the occipital lobe. In larger primates the IFOF’s location can be separated from the temporal middle and inferior longitudinal fascicles, but this becomes difficult in species where the temporal cortex is less gyrified. Therefore, we here placed a seed in the extreme capsule connecting temporal and frontal cortex and used an entire coronal section of temporal white matter at the level of the posterior commissure as target mask. A coronal exclusion mask encompassing the entire white matter except for the seed prevented spurious anterior–posterior tracts and a sagittal exclusion masks prevented leakage into the other hemisphere. Tractography was run with the seed and target masks and again with the roles of the two masks reversed and the results were averaged.

Tractography was performed using probtrackx2, using the default parameters except for the step length (0.15 mm for all samples apart from 0.1 for the galago). 1000 samples were seeded in each seed voxel. The resulting tractograms were normalized with respect to the total number of valid streamlines generated to allow comparison between species.

## Results

**Fig. 2:**
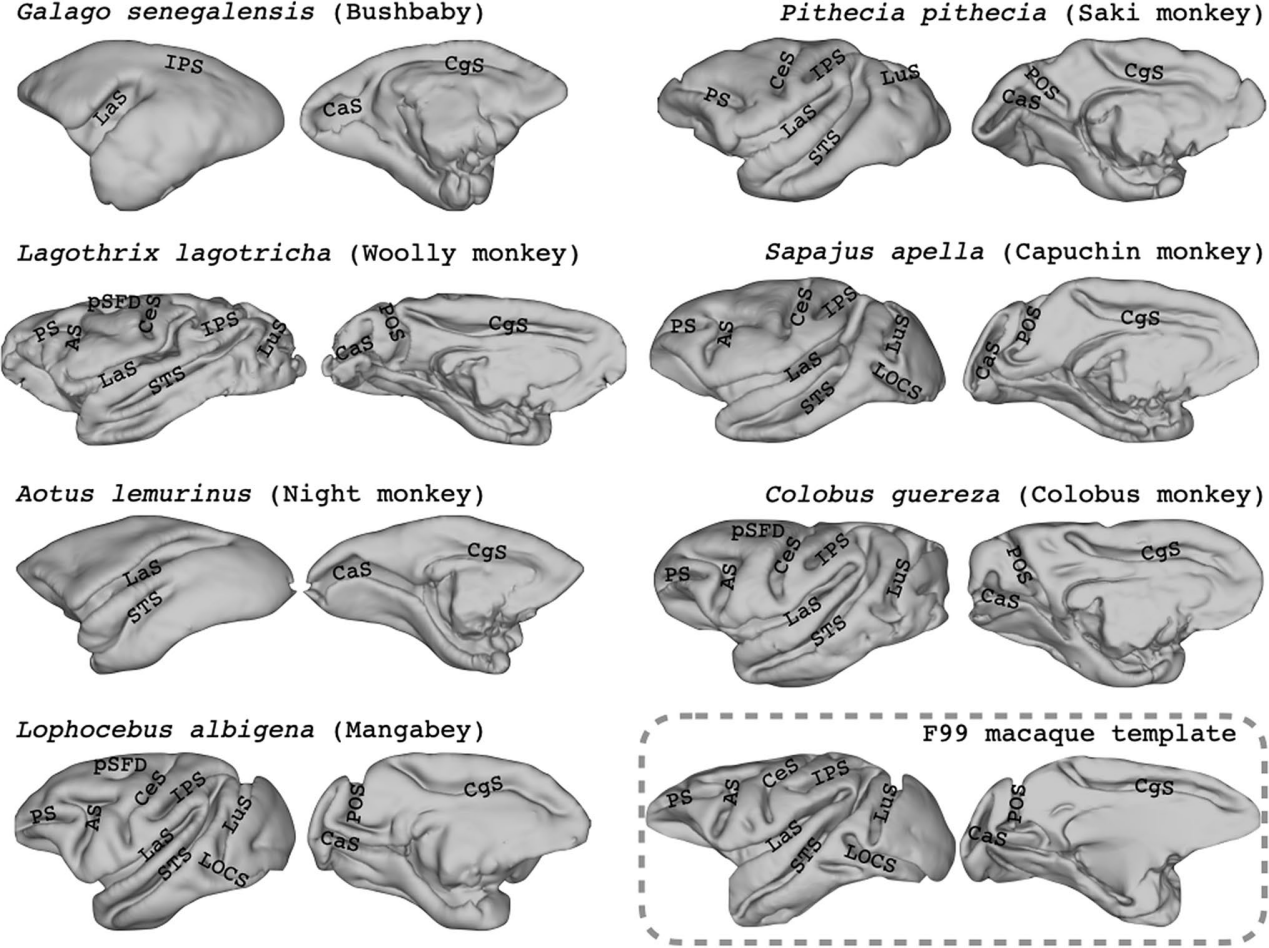
Preliminary sulcal labelling on the lateral surface of the cortical hemisphere of the prosimian and monkey species in this study. All surfaces were reconstructed from the scanned sample except for the macaque, for which we used the F99 template brain (Van Essen et al. 2012). Left hemisphere lateral sulcus on the left, medial surface on the right. *AS* arcuate sulcus, *CaS* calcarine sulcus, *CeS* central sulcus, *CgS* cingulate sulcus, *LOCS* lateral occipital sulcus, *IPS* intra-parietal sulcus, *LaS* lateral sulcus, *LuS* lunate sulcus, *POS* parietal–occipital sulcus, *PS* principal sulcus, *pSFD* posterior superior frontal dimple, *STS* superior temporal sulcus. Note that brains are not displayed to scale

We collected high-quality diffusion and structural MRI data from eight primate species that are not or little described in the comparative neuroimaging literature, as well as presenting data from the well-studied rhesus macaque. Samples were selected for their high quality and the absence of many large cuts due to the brain extraction process. Nevertheless, some cuts were present and evident in the imaging data around the occipitotemporal cortex in the night monkey, capuchin monkey, woolly monkey, and colobus monkey (Fig. 2). In addition, the saki monkey does not have a cerebellum attached anymore.

### Sulcal anatomy

Figure 2 shows a lateral view of a midthickness surface for the scanned prosimian and monkey samples and the macaque F99 surface (Van Essen et al. 2012) for comparison. We did not label the chimpanzee, as this great ape brain’s morphology is very different from the (pro)simian samples and a comprehensive atlas was recently published (Falk et al. 2018). Sulci were labelled predominantly using terms from the rhesus macaque or, if those were not present, the description of Connolly (1950) as a guide. As described before, the galago cortex shows very little gyrification, with the lateral and intra-parietal sulci as the only prominent sulci on the lateral surface and only dimples present in other locations. The night monkey shows only moderately more gyrification, with a quite extended lateral sulcus and a temporal longitudinal sulcus.

In the frontal cortex, most monkeys show a clear principal sulcus [previously termed the sulcus rectus, Connolly (1950)] and, except for the saki and night monkeys, a clear arcuate sulcus. The relative location of the arcuate sulcus and the presence of a spur at the back of it are variable, with some of the larger brains showing a clear posterior superior frontal dimple. On the medial wall, a cingulate sulcus running above the corpus callosum is evident in all species. However, the marginal sulcus is not present in the galago and only to a very limited extent in the night monkey.

The complexity of the occipital–temporal area also differs substantially across species, ranging from no gyrification in the galago, little in the saki monkey, to clear lunate and inferior occipital sulci in the capuchin, mangabey, and macaque. The intra-parietal sulcus is generally present, but its relationship with the posterior ends of the lateral and superior temporal sulci is variable. The most complex posterior gyrification can be seen in the woolly monkey, with distinct segments where the intraparietal sulcus is in Old World monkeys.

### White matter anatomy and tractography

**Fig. 3:**
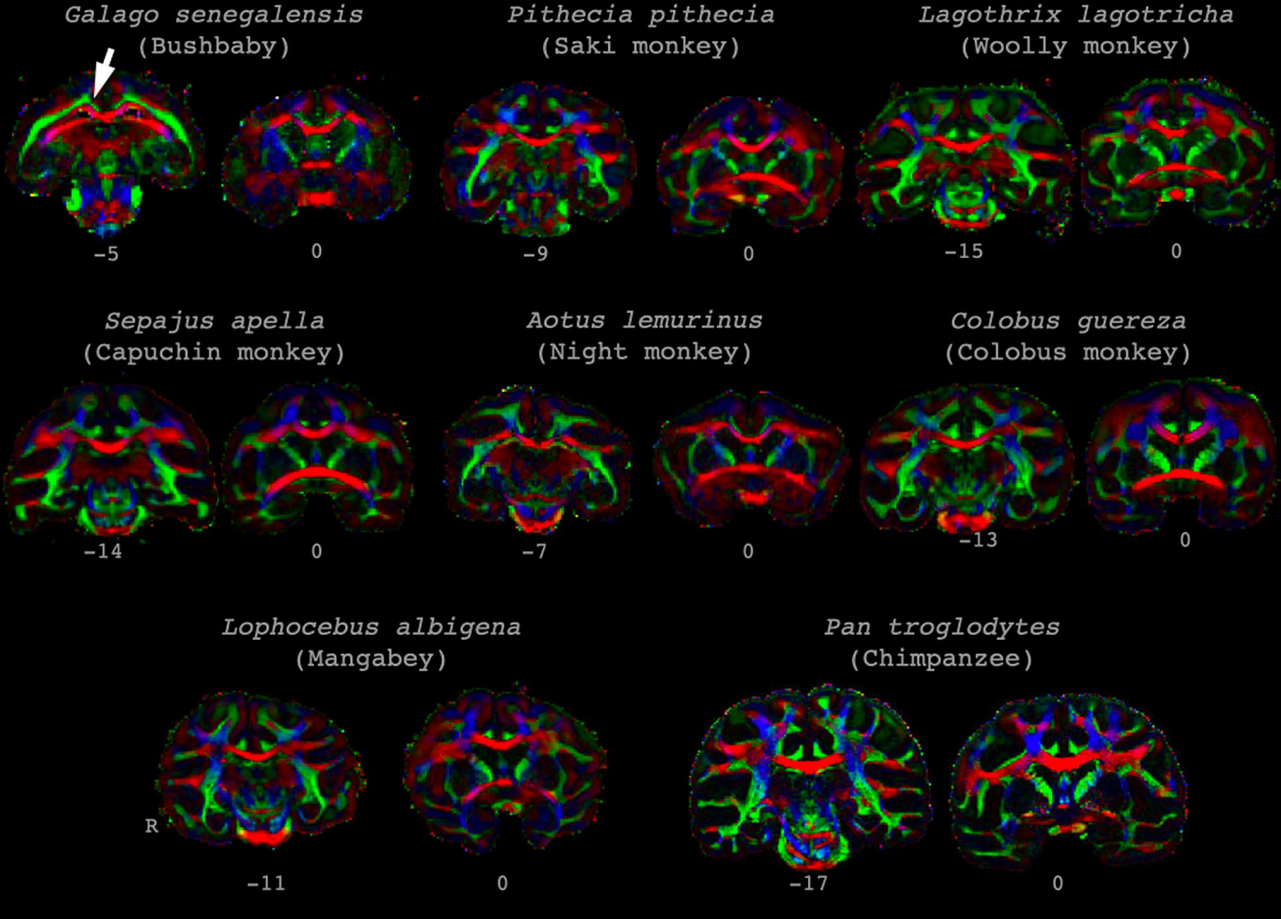
Principal diffusion direction maps. Images show coronal sections of the samples in this study at the level of the anterior commissure (*y* = 0) and the level approximately 25% from the posterior edge of the corpus callosum. As can be seen, the temporal white matter of bushbaby consists of a single sheet, rather than the more extensive pattern seen in more encephalized species, including the woolly monkey. Similarly, in the bushbaby and night monkey, the cingulum bundle consists of a white matter sheet that is difficult to distinguish from the superior longitudinal fascicle complex, as indicated for the bushbaby by the white arrow. Colors indicate principal directions, red left–right, green anterior–posterior, blue dorsal–ventral. Note that brains are not displayed to scale

We were able to obtain high-quality diffusion MRI data in all species. The pattern of orientation of the principal diffusion direction allows us to identify some of the prominent landmarks of the primate white matter (Fig. 3). Between hemispheres, the corpus callosum is the most prominent bundle, but the anterior commissure was also very present and could be used as the origin of the image. The posterior commissure was sometimes more difficult to identify, but can be used to orient the images to approximate AC/PC orientation. Prominent projections fibers, especially the upcoming fibers of the corticospinal tract and internal capsule were clearly present. In addition, anterior–posterior longitudinal fibers were clearly visible, even in areas of crossing fibers where they were not evident in the primary diffusion direction.

**Fig. 4:**
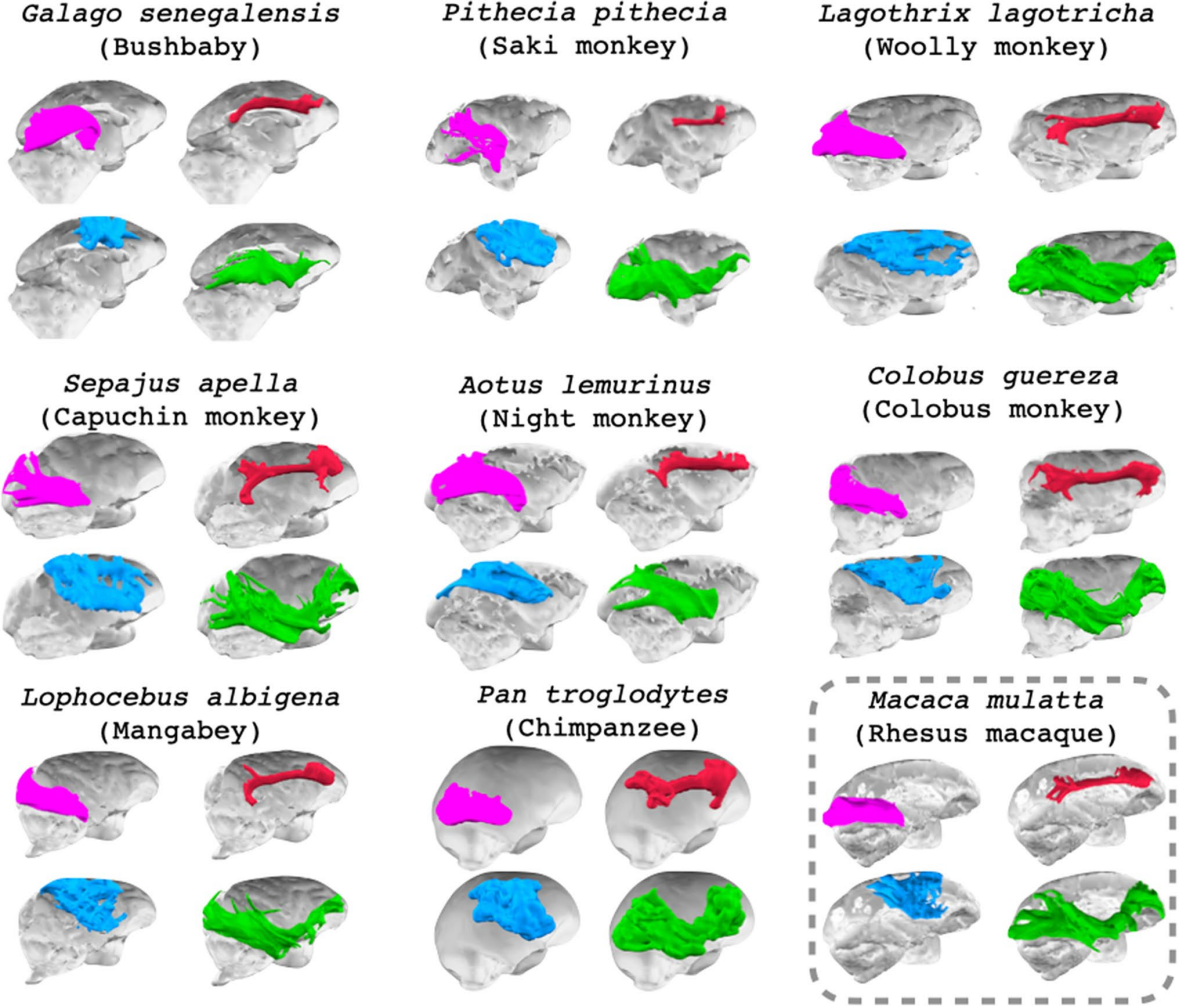
Course of reconstructed white matter tracts. 3d representations of the course of cingulum bundle (red), optic radiation (green), longitudinal fronto-temporal tract (blue), and SLF complex (purple) through the brains of all studied species

As a first reconstructed tract, we identified the optic radiation, a projection fiber tract carrying fibers from the LGN toward the primary visual cortex. This tract was identified in all species (Fig. 4). The same was true for the dorsal part of the cingulum, a longitudinal fiber bundle running just above and along the length of the corpus callosum. However, while this fiber tract is generally a separated bundle running through the cingulate gyrus, in the smaller brains, including the galago and night monkey, it consisted of a white matter sheet that was continuous with parts of the superior longitudinal fibers (Fig. 3).

The superior longitudinal fascicles (SLF) run dorsally between the parietal and frontal cortex. These fibers run through territory with multiple crossing fibers and as a result can be difficult to reconstruct (Behrens et al. 2007; Mars et al. 2019). Indeed, parts of the SLF can be seen in the secondary fiber directions of all species, including the galago. We here sought to establish whether we could reconstruct longitudinal tracts between parietal and frontal cortex in all species. To increase the sensitivity, we aimed to reconstruct the entirety of the SLFs as a single ‘complex’, rather than distinguishing the individual subbranches as is often done in more gyrified species, including the human and macaque (Schmahmann and Pandya 2006; Thiebaut de Schotten et al. 2011). Figure 4 shows that we could identify such tracts in all species.

In the human brain, a prominent fiber bundle termed the inferior fronto-occipital fascicle (IFOF) travels through the extreme capsule, connecting parts of occipital and temporal cortex with the frontal cortex. In more gyrified species, the IFOF can be distinguished from the middle and inferior longitudinal fascicles (MdLF, ILF) running in the gyri of the temporal lobe. However, in the prosimian galago the temporal white matter consists primarily of a single sheet making it difficult to distinguish sub-tracts. We therefore reconstructed what we termed a longitudinal frontotemporal tract (LFT) that should pick up the IFOF, but might include sections of MdLF, ILF, and uncinate fascicle, by selecting the entire coronal section of the temporal white matter in the target mask. We did keep the constraint of fibers having to pass through the extreme capsule to be included in the tractogram; this was done to make it more likely we isolated fibers belonging to the IFOF. We observed such a fiber running between frontal and occipitotemporal cortex in all species studied here, including the prosimian galago. In the galago, the bulk of the tractogram runs in the middle part of the temporal white matter sheet, which is consistent with the location of the IFOF in other species.

**Fig. 5:**
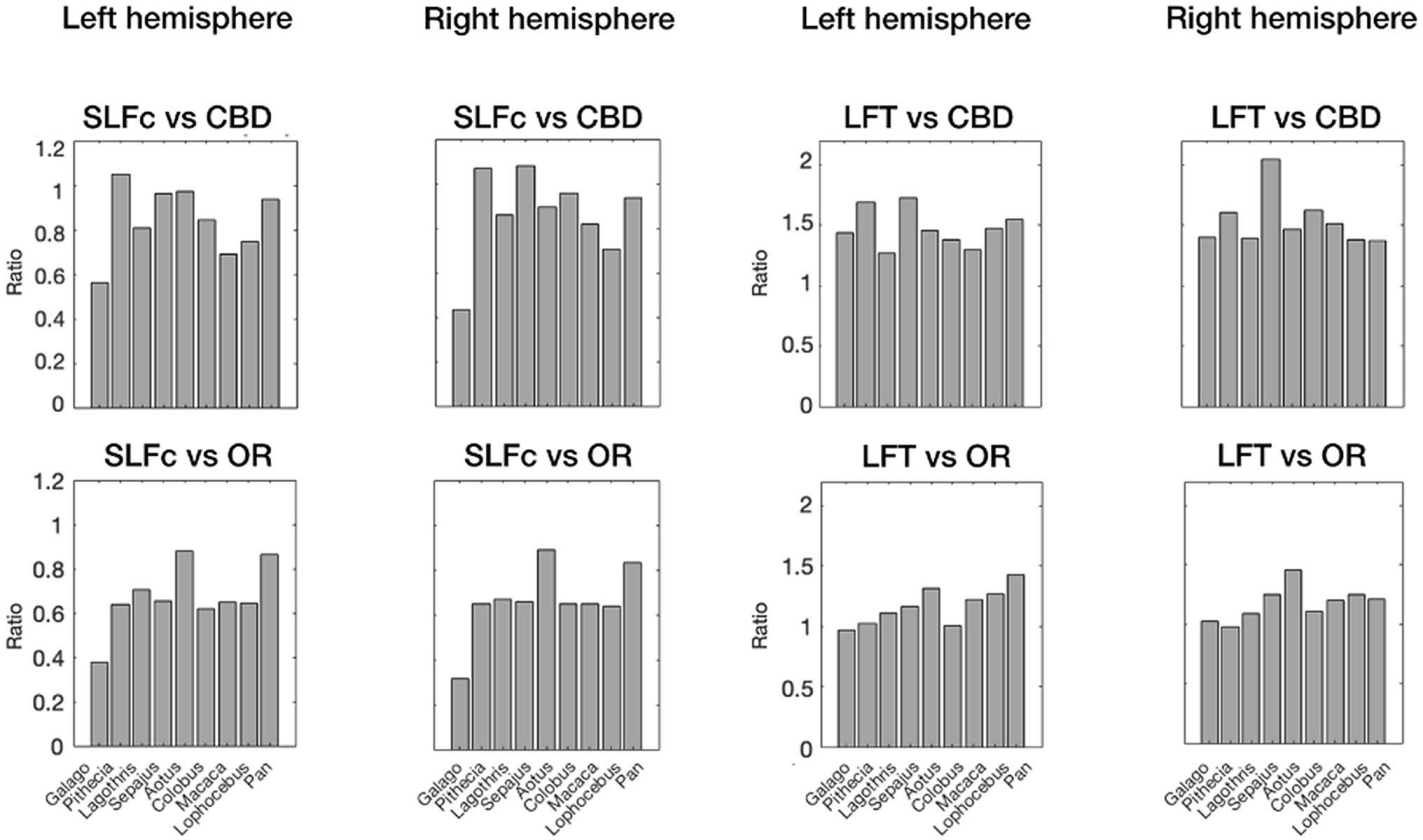
Relative size of the SLF complex (SLFc) and longitudinal frontotemporal tract (LFT) in all studied species. Relative sizes of SLFc and LFT are displayed with respect to the dorsal cingulum bundle (CBD) and optic radiation (OR) for both hemispheres

The longitudinal frontotemporal tract and the SLF complex are association fibers, connecting parts of association cortex that have preferentially expanded in some parts of the primate lineage (Chaplin et al. 2013). As such, one might expect these fibers to occupy more of the cortical white matter in anthropoid primates than in prosimians. To account for the size of the brain itself, we compared the volume of the association tracts with those of the non-association tracts (Fig. 5). This showed, albeit qualitatively, that the SLF complex of parietal–frontal fibers was least elaborate in the galago compared to all other primates. The same relationship was not observed for the fronto-temporal fibers.

## Discussion

Gaining a full understanding of the similarities and differences across different species’ brains requires multiple types of data that are available in multiple species, allowing comparisons across different scales within a species followed by comparisons between species using the same data types. We have termed these comparisons vertical translations and horizontal translations (Mars et al. 2021), respectively, and demonstrated that both are necessary to gain a full understanding of brain diversity (Eichert et al. 2020). Here, we present data on sulcal morphology and white matter anatomy from eight primate species that are not or hardly described in the literature. The data are freely available to allow quantitative comparisons across species. Importantly, the brain samples are still available at the Primate Brain Bank, opening the door for acquisition of more, including potentially more destructive, data types from these brains to increase the number of possible comparisons.

Sulcal labelling in the samples presented here is based on similarity in morphology and location of the sulci. Mostly, we based our terminology on labels commonly used in the macaque, supplemented by descriptions by Connolly (1950). Overall, the major sulci across the Old World monkeys are reasonably consistent. There appears to be more variability across species in the New World monkeys, with the night monkey showing the most simplistic morphology and the woolly monkey the most complex. The latter’s parietal sulcal pattern in particular is quite extensive. Interestingly, the woolly monkey possesses the largest brain of the New World monkeys, potentially speaking to debates of whether gyrification is mostly driven by brain size differences (Mota and Herculano-Houzel 2015; Amiez et al. 2021).

Given the limited information available for most of the species studied here, the proposed sulcal labels at this stage are not sufficient to prove that the sulci are the same across species. Indeed, whether sulcal patterning across species can be used for meaningful comparison is still a matter of debate (Amiez et al. 2019; Heuer et al. 2019), although relationships between sulcal anatomy and functional localization across individuals seem quite robust (Amiez et al. 2006; Eichert et al. 2021). Building on such results, recent work has used similarity in sulcal morphology to directly compare brains (Auzias et al. 2013) and show how it can be used to compare brains on the assumption that the sulci are directly comparable across species (Coulon et al. 2018). However, whether this assumption is justified needs additional testing. For instance, if one could establish similarities of cortical territories across multiple modalities, such as for instance their location in relation to prominent sulci, connectivity fingerprints, and cytoarchitecture, one can gain greater confidence in suggesting similarity of a given part of the brain across species (Mars et al. 2021).

Connectivity has been used to compare species quite extensively and the diffusion MRI data presented here can be used in this endeavour. This can be done by creating summary measures that describe, for instance, the efficiency of brains’ wiring diagrams (Assaf et al. 2020) or by comparing the network organization of different brains (van den Heuvel et al. 2016, 2019). Additionally, one can use these data to reconstruct specific white matter tracts that, in turn, can be used to compare brain organization across species by creating whole brain white matter tract connectivity blueprints (Mars et al. 2018). We have here created four of such tracts in first instance, using recipes that are maximally similar across species and can therefore be meaningfully compared.

The cingulum bundle is a limbic tract carrying fibers from the medial temporal lobe, curving around the splenium of the corpus callosum, running along the white matter of the cingulate gyrus and terminating in the frontal, including subgenual, cortex (Catani et al. 2013). Recent tract tracing work in the macaque showed that many of the projections running through this fiber system only travel through part of the tract, leading to a definition of three subdivisions (Heilbronner and Haber 2014). We here reconstructed the dorsal subdivision that runs through the cingulate gyrus white matter along the corpus callosum. This tract is expected to scale with the size of the brain, as limbic fibers are generally thought to be quite conserved across primates (Folloni et al. 2019). Similarly, the optic radiation is thought to be quite conserved across different primates (Catani et al. 2003), making it a suitable reference tract.

We reconstructed two association fibers. We were able to reconstruct longitudinal parietal–frontal tracts in all species. The SLF is generally divided into three distinct bundles (Petrides and Pandya 1984), which in turn are separate from the arcuate fascicle that projects more ventrally (Petrides and Pandya 1988). However, as a first approximation and to establish our ability to reconstruct the SLFs, we here grouped them together in an ‘SLF complex’. Comparison of this complex with the cingulum bundle and optic radiation shows that it is least extensive in the prosimian galago compare to other, anthropoid, primates. This is consistent with the so-called ‘anthropoid elaboration’ in cortico-cortical connectivity between parts of association cortex (Krubitzer 2009). Importantly, however, the SLF complex was present in the galago, consistent with tracer studies showing qualitatively similar parietal–frontal organization in galagos compared to other primates (Stepniewska et al. 2009; Kaas et al. 2011). Whether the anthropoid elaboration is a feature that can be predicted based on the size of the brain or whether if reflects a grade change between prosimian and simian primates is a matter that will require analysis in a full phylogenetic framework to address.

The presence of an inferior fronto-occipital tract (IFOF) or extreme capsule fiber complex has been accepted in the human brain for some time (Makris and Pandya 2008; Forkel et al. 2014), but its existence in the monkey has been controversial, due to the differential course of the tract suggested by tract tracing studies and some negative results using tractography in the macaque (Schmahmann and Pandya 2006; Takemura et al. 2017). Other authors, however, suggested a tract similar to the human IFOF could be identified in the macaque brain (Mars et al. 2016) when using similar tractography protocols in humans and macaques. More recently, dissection studies have confirmed the existence of a large white matter bundle along the course of the IFOF in both macaque and vervet monkeys (Decramer et al. 2018; Sarubbo et al. 2019). Here, we show the existence of an IFOF-like tract in all studied samples, including a frontal–occipital tract running medially in the temporal white matter sheet of the prosimian galago. Interestingly, a recent large-scale tractography study that included animals outside the primate order showed the presence of a ventral longitudinal tract across the mammalian order, although the lack of homologous temporal cortex in non-primate species means it is as yet unclear whether these tracts are homologous (Assaf et al. 2020).

The current results should of course be treated with caution. Although all animals were adults and the samples were selected for their high quality, the study is limited by its sampling range. More samples per species will allow one to address questions about within-species interindividual variability. A difficulty in increasing the sample number is the quality of the preserved brains required for this analysis. Brains should not be frozen and preserved with 24 h of death (D’Arceuil and de Crespigny 2007). Post-mortem cuts often made during brain extraction are making this endeavour more difficult as superficial cuts could impact on structural brain analysis (e.g., deformation-based morphometry) and deep cuts will interfere with the tractography analysis.

As indicated above, a full analysis of these data would ideally be done within in a formal phylogenetic framework (Harvey and Pagel 1991; Barton and Venditti 2013). Such analyses require quite extensive datasets. The current dataset adds to the growing number of initiatives that make data from a variety of species openly available to the scientific community (Thiebaut De Schotten et al. 2019). Such initiatives include the Primate Data Exchange (Milham et al. (2018); fcon_1000.projects.nitrc.org/indi/indiPRIME.html); the National Chimpanzee Brain Resource (www.chimpanzeebrain.org), the Japan Monkey Centre Primates Brain Imaging Repository (Sakai et al. (2018); j-monkey.jp/BIR/about_e.html); resources focused specifically on the marmoset (e.g., Liu et al. (2021), marmosetbrainmapping.org/data.html; Woodward et al. (2018), https://www.brainminds.riken.jp/atlas-package-download-main-page); and the brains provided in Heuer et al. (2019) and Navarrete et al. (2018). The latter of these contains structural images from the Primate Brain Bank, the same sample source as used in the present manuscript. In a parallel effort, resources for analysing data from non-human primate are also increasingly shared openly and support fora are emerging, including the Primate Resource Exchange (Messinger et al. 2021). These resources provide great opportunities for comparative science, and have already inspired and enabled projects of larger scale than possible by any one laboratory on its own.

## Data Availability

The pipeline for processing the spin-echo data uses FSL (www.fmrib.ox.ac.uk/fsl) and is implemented in the MR Comparative Anatomy Toolbox (Mr Cat; www.neuroecologylab.org) as the ‘phoenix’ module. The surface reconstruction pipeline is available at https://github.com/ardesch/nhp-freesurfer. All data are made available for scientific purposes on the WIN Digital Brain Bank (https://open.win.ox.ac.uk/DigitalBrainBank/) subject to a Material Transfer Agreement. Tractography and results are available as a Data Sharing Collection link for reviewers: (https://data.donders.ru.nl/collections/di/dcc/DSC_2020.00034_408).
